# DINOSAUR: an integrated cognitive-behavioral treatment for anxiety in young children with ASD

**DOI:** 10.1186/s11689-021-09396-9

**Published:** 2021-10-11

**Authors:** Amy Keefer, Roma A. Vasa

**Affiliations:** 1 Center for Autism and Related Disorders, Kennedy Krieger Institute, Baltimore, USA; 2 Department of Psychiatry and Behavioral Sciences, Johns Hopkins University School of Medicine, Baltimore, USA

**Keywords:** Autism, Anxiety, Treatment, Young child

## Abstract

**Background:**

Anxiety disorders are highly prevalent in children and adolescents with autism spectrum disorder and often emerge before the age of 6 years. Yet, only a few studies have examined anxiety treatment for this group. Preliminary evidence from these studies suggests that utilizing cognitive behavioral therapy (CBT) as well as strategies to target intolerance of uncertainty (IU) and parental accommodation, known mechanistic and maintaining factors of anxiety may improve anxiety and optimize outcomes in this age group.

**Main body:**

To meet this need, we developed an integrated treatment called DINO Strategies for Anxiety and intolerance of Uncertainty Reduction (DINOSAUR), a 12-week group telehealth treatment for 4- to 6-year-old children with ASD. DINOSAUR works with young children and their parents to deliver CBT along with interventions targeting IU and parental accommodation. In this paper, we first discuss the rationale for developing this treatment and then describe a pilot study of its feasibility and preliminary efficacy.

**Conclusions:**

There is a great need to develop anxiety treatments for young children with ASD. We proposed a novel integrated treatment approach that aims to alter the way young children and parents respond to fear, which could potentially improve short- and long-term mental health outcomes for this age group.

**Trial registration:**

ClinicalTrials.govNCT04432077 on June 03, 2020

## Background

Co-occurring psychiatric conditions are highly prevalent in children with autism spectrum disorder (ASD), with anxiety disorders being one of the most common conditions in this population [41]. Approximately 20% of children under 6 years and 40% of children ages 6 to 18 years with ASD have one or more anxiety disorders [45, 47]. Children with ASD and co-occurring anxiety have increased rates of self-injurious behavior, depressive symptoms, and parental stress compared to children with ASD without anxiety and are also at increased long-term risk for other psychiatric disorders [22, 34]. Hence, developing effective anxiety interventions for children with ASD, especially when they are young and neuroplasticity is heightened, is a top priority in the field.

Over the past decade, the field has made great progress in developing cognitive behavioral therapy (CBT) models for children with ASD and co-occurring anxiety; however, these studies have primarily targeted school-age children, ages 7 to 14 years of age with average or above average cognitive levels. Fourteen randomized-control and 2 open-label trials of CBT, modified for school-age children with ASD, support its efficacy in reducing anxiety in this age group, with most studies reporting medium effect sizes and remission rates ranging from 38.0% to 71.4% [44, 46]. Only four anxiety treatment studies have been conducted in children under 6 years old, of which three utilized CBT approaches [3, 8, 9, 26]. Preliminary findings from these studies suggest that CBT may be effective for this age range; however, further study is needed. Results also suggest that targeting intolerance of uncertainty (IU) and parental accommodation, known mechanistic and maintaining variables, respectively, of anxiety in neurotypical and ASD populations, may be effective in reducing anxiety [12, 18, 20].

There is mounting evidence supporting the need to target IU and parental accommodation when treating anxiety in children with ASD. IU is the “dispositional incapacity to endure the aversive response triggered by the perceived absence of salient, key, or sufficient information” ([5], p. 31). Children with ASD have higher levels of IU than neurotypical children, and preliminary evidence suggests that IU may mediate the relationship between ASD and anxiety [4, 48]. Parental accommodation refers to parental behaviors that attempt to prevent or reduce child distress when the child is participating in age-appropriate activities and experiences fear or anxiety [25]. This can include behaviors such as providing excessive reassurance and allowing children to avoid stimuli that make them anxious, which reinforce the child’s avoidance and prevent opportunities for corrective learning [43]. Parental accommodation is highly prevalent in families of children with anxiety and ASD and has been identified as an important maintaining variable of anxiety [12, 43]. Targeting IU and parental accommodation in the context of CBT has the potential to alter maladaptive fear response in the child and parent, thereby reducing long-term risk of psychiatric disorders.

The purpose of this article is to describe the development of a novel, integrated anxiety treatment model, DINO Strategies for Anxiety and intolerance of Uncertainty Reduction (DINOSAUR), for young children with ASD (i.e., those under 6 years old). This treatment is integrated because it utilizes CBT components that have been adapted for both ASD and young children; targets IU, an underlying mechanistic construct; and places heavy emphasis on reducing parental accommodation. In this paper, we will first describe the rationale for developing DINOSAUR by reviewing the existing anxiety treatment literature for young children with ASD and by discussing neurodevelopmental factors that we considered when designing a treatment for this age group. We will then describe our pilot study examining the feasibility and efficacy of DINOSAUR including the treatment’s structure and components.

## Anxiety treatment studies in young children with ASD

Most of the anxiety treatment studies in children with ASD under 6 have studied models of CBT. CBT is a well-established, first-line treatment for anxiety in neurotypical children [16]. The core components of CBT for anxiety include psychoeducation about anxiety, restructuring of anxious thoughts, relaxation practice, and graded exposure to feared stimuli [33]. In ASD, these components are modified to accommodate the various social-emotional, language-based, and neuropsychological challenges of this population. Specific modifications for school-age children with ASD focus on improving emotion recognition, reducing abstract language, simplifying tasks that require metacognition such as cognitive restructuring, and incorporating strategies to engage the child and generalize skills to real world settings [30]. When these treatments are delivered to young children with ASD, additional modifications are needed to account for their early developmental, socioemotional, and neurocognitive profiles.

To date, a total of four studies have targeted anxiety in young children with ASD (See Table 1). Two of the studies examine the efficacy of two CBT protocols, *Being Brave* and *Fun with Feelings*, for children who are actively experiencing either anxiety disorders or symptoms [8, 9]. A third study focuses on the prevention of anxiety using the *Cool Little Kids* CBT protocol in children with behavioral inhibition [3]. All three of these treatments include adaptations that account for the developmental level of the young child [3, 8, 9], but only one includes adaptations specifically for children with ASD [8]. The fourth study investigates changes in anxiety following a naturalistic developmental behavioral intervention for ASD, *Pivotal Response Training* [26]. None of these studies target intolerance of uncertainty, and only one directly targets parental accommodation.

**Table 1:** Anxiety treatment studies for young children with autism spectrum disorder (ASD)

Intervention	Participants	Treatment	Format	Results
[9]	3–7-year-old children with ASD and anxiety disorders; IQ ≥ 80	CBT *Being Brave* (*n*=16)	6 parent training sessions 7 parent-child sessions	Significant decreases in anxiety with 40% and 82% remission rates of any anxiety disorder at post-treatment and 4-month follow-up, respectively^a^
[8]	4–6-year-old children with ASD and clinically significant anxiety; absence of ID^b^	mCBT^c^ *Fun with Feelings* (*n*=31)	10 parent-only sessions	No change in anxiety symptoms at post-treatment or at 3-month follow-up^d^
[3]	4-year-old children with ASD and behavioral inhibition; IQ not assessed	CBT *Cool Little Kids* (*n*=26)	6 parent-only sessions	Active treatment group had a significantly lower rate of anxiety disorders compared to controls at 1-year follow-up^e^
[26]	4-8 year-old children with ASD IQ ≥ 70	*Pivotal Response Treatment* *(n=21)*	48 sessions over 16 weeks (5h of child intervention; 2h parent training per week)	Children displayed a significant reduction in anxiety and internalizing symptoms after treatment^f^

^a^Outcomes assessed using the Pediatric Anxiety Rating Scale [49], Spence Preschool Anxiety Scale [42], Child Behavior Checklist [2], and Kiddie Schedule for Affective Disorders and Schizophrenia for DSM-IV – Epidemiologic Version [32]

^b^Intellectual disability

^c^Modified cognitive behavioral therapy for ASD

^d^Outcomes assessed using the Pediatric Anxiety Rating Scale [49] and Child Behavior Checklist [2]

^e^Outcomes assessed using the Anxiety Disorder Interview Schedule for DSM-IV-Parent Interview Schedule [40]

^f^Outcomes assessed using the Child and Adolescent Symptom Inventory and Child Behavior Checklist [13]

When evaluating the two CBT protocols that target anxiety, similarities and differences are present in both the components and the format of treatment. Both protocols include the core CBT components of psychoeducation, coping skills, and exposure and were deemed feasible and acceptable by participating parents [8, 9]. *Being Brave* is a parent-child anxiety treatment that was originally designed for school-age, neurotypical children and adapted for a younger population [9, 17, 19]. The treatment consists of 13 sessions that focus exclusively on targeting anxiety symptoms. In contrast, *Fun with Feelings* is a 10-session, parent-only intervention adapted from a protocol designed to improve general emotional regulation in young children with ASD [8, 11]. Results from these preliminary trials showed that children participating in *Being Brave* exhibited significant decreases in anxiety, with 40% and 82% remission rates of any anxiety disorder at post-treatment and 4-month follow-up, respectively [9]. However, those in *Fun with Feelings* displayed no change in anxiety symptoms immediately following the intervention, though a significant decrease in internalizing symptoms was observed at 3-month follow-up [8]. Potential reasons for treatment response in the *Being Brave* protocol are the use of a parent-child versus a parent-only model, the longer treatment duration, and exclusive focus on anxiety versus anxiety plus emotion regulation.

The anxiety prevention treatment was developed for young neurotypical children and delivered without modifications for ASD [3, 35]. This treatment is exclusively a parent training model that teaches parents how to create exposure hierarchies targeting their child’s fears. The treatment also places heavy emphasis on targeting parental accommodation by increasing parents’ awareness and modification of their own anxious thoughts to avoid behaviors that may reinforce anxiety in their child. One year after treatment, children in the active treatment group had significantly lower rates of anxiety disorders and, 2 years later, a significantly lower rate of separation anxiety symptoms compared to a treatment-as-usual control group [3]. These findings suggest that targeting parental accommodation may be an important feature of anxiety treatment in this age range. One major piece of feedback from the parents was that the treatment needed to be more customized to address the unique features of young children with ASD [3].

The final study, which is not a CBT study, examined changes in anxiety as a secondary outcome following a trial of *Pivotal Response Treatment* (PRT; [26]). This intensive treatment targets the core social deficits of children with ASD by providing enticing social opportunities and natural reinforcement for appropriate social responses. Although PRT does not directly target anxiety, parents reported that their children displayed a significant reduction in anxiety after treatment. One reason for the improvement could be that PRT increases the predictability of social interactions, thereby reducing the child’s uncertainty and anxiety in these situations [26]. While effective, treatment duration was quite intensive, i.e., 48 sessions, which may not be feasible for some families.

Collectively, findings from these preliminary treatment trials provide a blueprint for designing future anxiety studies in young children with ASD. Specifically, findings from the CBT studies indicate that a parent-child treatment model may be effective, but further study is needed given that only one study has examined this model in this age group. Additionally, findings from the anxiety prevention and PRT studies indicate that targeting parental accommodation and reducing social uncertainty, respectively, may also reduce anxiety. Last, parent acceptability data indicate that it is important to customize the intervention for young children with ASD. DINOSAUR incorporates each of these factors to create an integrated anxiety treatment that targets anxiety, IU, and parental accommodation.

## Neurodevelopmental considerations when designing DINOSAUR 

We considered both child- and parent-specific factors when designing the DINOSAUR treatment. Young children with ASD often have difficulty responding to social cues, become overfocused on specific objects or interests, display deficits in social imitation, experience difficulty adapting to new routines or expectations, and have short attention spans, immature executive functioning, and limited abstract reasoning [28, 37]. DINOSAUR addresses these various challenges in three ways. First, the treatment is centered around an engaging theme, dinosaurs, which is a preferred interest of many young children with ASD. Each component of the program is presented to participating children using dinosaur-themed images and activities. Second, the treatment utilizes empirically based educational strategies that have been shown to be effective for enhancing learning in young children with social-emotional impairments. These include the use of video- and peer-based modeling to teach new skills, frequent rehearsal to reinforce concepts and strategies, the integration of visual cues throughout the program, and the use of weekly visual schedules and consistent routines to start each session [28]. Last, to address the neurocognitive profiles of this age group, treatment sessions involving the child are short (e.g., 20–35 min) and include movement-based activities, concrete strategies, and animated and engaging therapeutic materials (e.g., story books and stuffed animals).

Parents of young children with ASD also face unique challenges that should be considered when designing an anxiety intervention for this age range. As most parents have only recently received their child’s ASD diagnosis, their understanding of the core symptoms of ASD and how it presents in their child is often not fully developed. This can complicate their ability to differentiate anxiety from ASD and from normative anxiety that is consistent with typical development. To address this, an entire session in DINOSAUR is dedicated to teaching parents how to differentiate anxiety from ASD symptoms, and to carefully consider whether anxiety is excessive and causing impairment. Another challenge faced by parents of young children with ASD is the time commitment involved in having their child participate in multiple and often intensive intervention services for core ASD symptoms. To meet this need, DINOSAUR is delivered via a telehealth format so that parents and children can participate from their homes, thereby eliminating the time and stress of commuting to and from the clinic. The telehealth format also reduces the stresses associated with transitioning their young child from the home and finding childcare for siblings. The opportunity to conduct treatment sessions with families in their homes provides the added advantage of observing the parent and child in their natural environment.

## Pilot study of DINOSAUR

### Study overview and aims

This pilot study is a 12-week, CBT waitlist controlled treatment for anxiety in 4- to 6-year-old children with ASD. The treatment is group-based, includes parents and children, and targets anxiety, IU, and parental accommodation. All study procedures (e.g., consent, anxiety and ASD assessments, anxiety treatment) will be conducted via phone or a secure videoconferencing platform to comply with current COVID-19 restrictions. This study is funded by the Organization for Autism Research and a private donor. All procedures were approved by the Johns Hopkins Internal Review Board.

The study has two aims. The primary aim is to determine the feasibility and acceptability of the DINOSAUR treatment model. We hypothesize that DINOSAUR will be feasible and acceptable based on rates of participation, measures of parental satisfaction, and parents’ acquisition of key intervention strategies. The second aim is to examine the preliminary efficacy of DINOSAUR. We hypothesize that levels of anxiety, IU, and parental accommodation will show a significantly greater reduction following the DINOSAUR intervention than after a 12-week, treatment-as-usual (TAU) waitlist condition. If these hypotheses are proven true, the next steps are to conduct a larger scale, randomized controlled trial of DINOSAUR.

### Participants

A total of 16 parent-child dyads will participate in the treatment program. Children will be included if (a) they are between the ages of 48 to 83 months, (b) have an IQ and language level ≥ 85, and (c) meet criteria for at least one of the following DSM-5 anxiety disorders (i.e., separation anxiety disorder, social anxiety disorder, generalized anxiety disorder) or the presence of clinically impairing anxiety presentations that are distinct in ASD (e.g., social anxiety without fear of negative evaluation; [21]). Children will be excluded if they have (a) a history of psychological trauma; (b) a history of neurologic illness; (c) history of participating parent substance abuse, bipolar disorder, or psychosis; (d) parents who are not English speaking; or (e) severe behavior challenges that prevent their participation in treatment groups (i.e., severe tantrums, aggression, or self-injury).

ASD diagnosis will be confirmed by either of two approaches: (1) an in-person evaluation at our autism center within the last year in which the child received a clinical diagnosis of ASD and met criteria on the *Autism Diagnostic Observation Schedule, Second Edition* [27] or (2) clinically significant scores on the *Autism Spectrum Rating Scales, Parent Rating Scale: Long Form* (ASRS; [14]) and structured observation via telehealth of the child’s social communication skills and behavior reviewed by a senior member of the study team with expertise in diagnosing ASD in young children.

IQ will be assessed by either of two approaches: (1) cognitive evaluation at our autism center within the last year utilizing any standardized cognitive assessment measure or (2) administration of the *Stanford-Binet Intelligence Scales, Fifth Edition (SB-5) Abbreviated Battery* [38] in concordance with the tele-neuropsychology guidelines offered by [15]. Expressive and receptive language level will be evaluated using the *Oral and Written Language Scales, Second Edition* [6].

Anxiety disorder status will be established by administration of the *Anxiety Disorder Interview Schedule with Autism Addendum* (ADIS/ASA [23];). The ADIS/ASA is comprised of (1) a semi-structured interview that assesses anxiety disorders in neurotypical youth [40] and (2) ASA supplement that facilitates the use of this tool in children with ASD [23]. The ADIS/ASA is the only anxiety measure for this population that strategically teases apart anxiety and ASD symptoms and measures both DSM-5 anxiety disorders and clinically impairing anxiety presentations unique to ASD. The ADIS/ASA has been further modified for young children by reminding testers to consider developmentally appropriate expectations of anxiety and behavior for this age group, providing parents with behavioral versus cognitive/verbal examples of anxiety, and asking about cultural factors and parenting styles that could affect anxiety presentations. Anxiety disorder status is determined by the clinician who assigns a Clinician Severity Rating (CSR) of 1 (not a problem) to 8 (severe impairment), with 4 being the cut-off for a disorder.

### Study procedures

Pilot data on DINOSAUR will be collected in two stages. The first is a pre-pilot study stage in which data are collected from 4 parent-child dyads who will participate in the 12-week treatment. Their data will be analyzed and used to refine the DINOSAUR intervention. Parents will complete measures of acceptability and clinicians will complete measures of treatment fidelity during the treatment. Within 2 months of treatment completion, parents will complete post-treatment assessments administered by an independent rater. Parents will also complete a final assessment to gauge parents’ understanding of key therapeutic concepts and participate in a qualitative interview about the DINOSAUR program. Throughout the study, parents will be asked to report any new interventions (e.g., therapy, medications, school services), as well as significant life events that the child may have experienced (e.g., family moves, start of school) to control for the potential effects of these factors on anxiety levels across time.

In the second stage, the pilot treatment will be delivered to three separate groups of four parent-child dyads. This stage will employ a waitlist control design in which each group will first participate in a treatment-as-usual (TAU) waitlist period for 12 weeks during which the child will maintain their current intervention programs and pursue new treatments as they wish. The group will then participate in the DINOSAUR intervention. Treatment outcome measures will be delivered at baseline (before participation in the waitlist period), pre-treatment (at the end of the 12-week waitlist period and before beginning the treatment), and post-treatment (following the 12-week intervention). Parents and clinicians will complete the same measures of treatment satisfaction and fidelity as completed in the first stage. The final group of parents will also participate in a qualitative interview about their experiences with the DINOSAUR program.

### Study measures

#### Measures to assess feasibility and acceptability (aim 1)

Feasibility will be evaluated in three ways. First, it will be assessed by measuring the proportion of the sample that was successfully retained through study completion and the percentage of treatment sessions attended by study participants. Second, feasibility will be evaluated by examining parents’ adherence to the intervention. This will be determined by comparing parents’ implementation of intervention skills during selected sessions to expected benchmarks determined by the study team before the start of treatment. Third, parents will complete a final evaluation assessing their knowledge of key treatment concepts.

Acceptability will be measured descriptively through parents’ and clinicians’ weekly ratings of treatment satisfaction. Parents in the first and final groups will also participate in a qualitative interview at the conclusion of the treatment to provide information about treatment perception, barriers, and satisfaction. Interviews will be audio-recorded and transcribed for analysis.

#### Measures to assess anxiety, intolerance of uncertainty, and parental accommodation outcomes (aim 2)

Four anxiety outcome measures will be included (categorical, dimensional, global improvement) in the study. These are as follows: (1) The ADIS/ASA CSR scores [23], which reflect the presence of clinically significant anxiety disorders or distinct anxiety presentations, will be the primary anxiety outcome measure and will be administered to parents at baseline, pre-treatment, and post-treatment. (2) The *Anxiety Problems* subscale of the *Child Behavior Checklist (CBCL/1.5-5 years* and *CBCL/6-18* [1, 2]) will be administered to parents at baseline, pre-, and post-treatment as a dimensional measure of anxiety. The *CBCL* is an established 99-item (*CBCL/1.5-5 years*) or 113-item (*CBCL/6-18 years*) parent-report measure of broad psychopathology that has been well validated in children with ASD [29]. (3) The *Preschool Anxiety Scale–Revised* (PAS-R [10]) will also be administered to parents at baseline, pre-, and post-treatment as a second dimensional measure of anxiety. The *PAS-R* is a 30 item parent-report measure of anxiety in preschool aged children that includes items assessing generalized anxiety, social anxiety, separation anxiety, obsessive-compulsive disorder, and physical injury fears and has been used in other anxiety treatment studies for children with ASD [9]. (4) The *Clinical Global Impressions Scale – Improvement* (CGIS-I [31]) will be administered to assess global improvement at the end of treatment. This is a 7-point scale completed by an independent evaluator reflecting the overall impression of treatment response based on information obtained through administration of the ADIS/ASA. Scores range from 1 (very much improved) to 7 (very much worse). A CGI-Improvement score of 1 (very much improved) or 2 (much improved) will be used to designate treatment response.

Intolerance of uncertainty will be assessed using the *Responses to Uncertainty and Environmental Structure Questionnaire* (RULES; [39]). The RULES is a 17-item parent-report measure that assesses response to uncertainty and low environmental structure in 3- to 10-year-old children. The RULES has been validated as a psychometrically sound measure of uncertainty in young children with anxiety [39]. Parental accommodation will be assessed using the *Family Accommodation Scale* (FAS [25]). This 9-point parent report scale assesses parental accommodating behaviors and has been previously used in child anxiety treatment studies [24]. These measures will be administered to parents at baseline, pre-, and post-treatment.

### Treatment model

#### Overview of the 12 sessions

The 12-week treatment consists of four 90-min parent-only sessions, followed by eight 60-min parent-child sessions. All sessions are led by two experienced clinicians (a psychologist and social worker) with extensive clinical and research experience in delivering CBT for the treatment of anxiety in children with ASD [18, 36]. Four core CBT components (i.e., psychoeducation, relaxation strategies, cognitive coping, and exposure) are delivered across the 12 sessions to target anxiety, IU, and parental accommodation. Participants complete two types of exposure hierarchies, one targeting anxiety and one targeting IU.

**Fig. 1:**
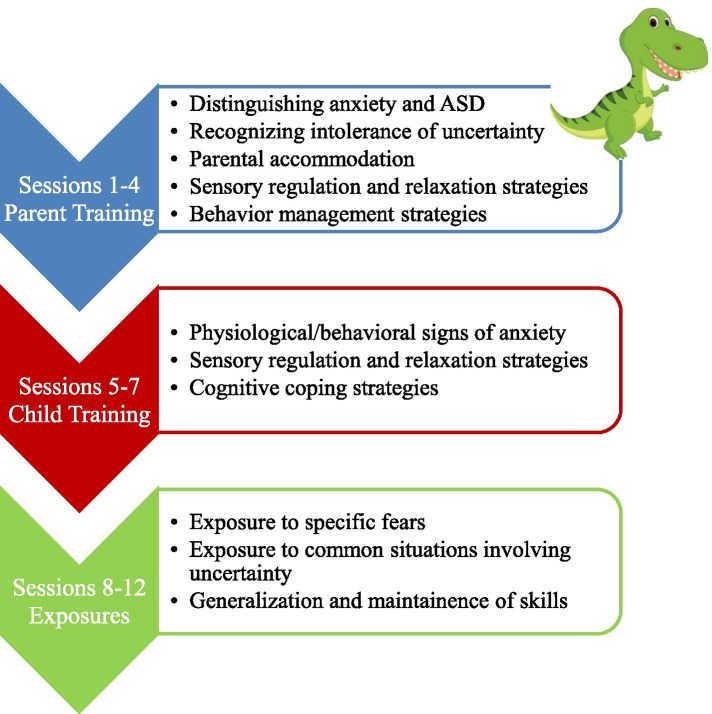
Treatment Overview

Figure 1 illustrates the DINOSAUR treatment model. The treatment is divided into three sections: parent training (sessions 1–4), child training (sessions 5–7), and exposure (sessions 8–12). Parent training sessions focus on education about anxiety, IU, and parental accommodation. Parents also learn each of the skills that will be taught to the child, including anxiety identification, relaxation strategies, and cognitive coping strategies.

Child training sessions involve working with parents and children together to teach the child about anxiety and IU, coping skills (i.e., relaxation and cognitive coping strategies), and the concept of exposure. Following completion of the child training activities, the children are dismissed. The remaining time is spent talking with the parents about how to address any difficulties that may arise as they direct their child’s home practice of new coping skills and how to implement exposure hierarchies targeting their child’s fears and IU.

The exposure sessions are dedicated to teaching parents and children how to implement two sets of exposures at home (e.g., targeting anxiety and IU) and how to help their child apply their new coping skills during those exposures. In the first half of each session, the group watches video recordings of fellow group members’ home exposure practice and provides reinforcement to the featured parent-child dyads. The children are then dismissed and parents are split into groups in which they receive feedback regarding their child’s exposure practice and plan the next week’s exposure steps. The last session focuses on reviewing key concepts and developing plans to continue implementation of anxiety management skills to maintain treatment gains and facilitate future exposures to address new fears.

#### Four core treatment components

The four core components of CBT utilized in DINOSAUR include psychoeducation, relaxation strategies, cognitive coping, and exposure. Psychoeducation is one of the hallmarks of DINOSAUR and is consistently delivered throughout the 12 sessions. The parent training section starts with a discussion of how to differentiate anxiety from ASD symptoms and how to recognize IU. Time is also spent discussing how parental accommodation can maintain anxiety, and parents are given techniques to manage their own anxiety about their child. Throughout the program, parents also receive explicit instruction about simple, behavioral management strategies to support their child’s success (e.g., ignoring anxious behavior, delivering reinforcement). During the child training sessions, psychoeducation about the symptoms of anxiety and the concept of exposure is provided through a series of stories about an anxious dinosaur, Nervosaurus Rex, that is read to the children. The children also complete simple worksheets to practice applying concepts introduced in the book to real life. Much of this work seeks to increase the child’s awareness of the physiological and behavioral signs of anxiety he/she most commonly experiences.

Another important treatment component of DINOSAUR involves teaching children and parents about relaxation skills. These include sensory-based calming activities, e.g., watching a repetitive image like water dripping, or listening to calming sounds like ocean waves, and structured relaxation strategies to manage anxiety and IU. Parents and children also jointly practice three structured relaxation skills, i.e., diaphragmatic breathing, progressive muscle relaxation, and yoga, which are simplified and presented using the dinosaur theme (e.g., children are taught to “roar like a dinosaur” when exhaling during diaphragmatic breathing). Parents are also encouraged to employ these skills to manage their own stress and anxiety.

Teaching basic cognitive coping skills is another component of the treatment, although these skills are utilized to a lesser extent than with older children with ASD due to the young child’s limited abstract reasoning and meta-cognitive skills. Parents and children are taught how to recognize the child’s “scared thoughts” which are distorted, unrealistic, and overly repetitive thoughts that may increase and maintain anxiety and IU. Parents are encouraged to help their child identify “helping thoughts” to replace “scared thoughts” and prompt their use. These can include short thoughts that are positive and empowering (e.g., “I can be brave.” when facing specific fears or “Surprises can be fun.” when confronting uncertainty), or fact-based thoughts that apply directly to the child’s specific fear (e.g., “Tornados don’t usually happen where I live.”). Parents are also encouraged to offer their child thoughts that relate to the dinosaur theme (e.g., “I am brave and strong like a T-Rex!”) or other preferred interests.

The final and most important component of the intervention is exposure. Unlike other CBT models, children in the current treatment model complete two types of exposures, one targeting feared stimuli that are part of their primary anxiety disorder and others targeting situations in which the child encounters uncertainty. For the first type of exposure, parents work with the clinicians to develop and implement a graded hierarchy for that fear. For the second type, parents select from several templated hierarchies involving uncertainty in daily life that are often difficult for young children with ASD (e.g., encountering social situations or completing tasks in which the expectations are uncertain). During exposures, children are cued to utilize their newly acquired coping skills and reinforced for completing each step in the hierarchy. Parents are also encouraged to manage their own anxiety and reduce accommodation during their child’s exposure using coping strategies learned in previous sessions. In session 12, parents work with the group leaders to develop additional exposures that may be helpful to address their child’s anxiety and IU following the conclusion of the group.

## Limitations and next steps

While DINOSAUR advances treatment for anxiety in young children, there are several limitations of this new treatment model. The first is that the treatment focuses on treating anxiety in children with average or above average cognitive and language skills. As approximately 45% of children with ASD experience co-occurring intellectual disability, it will be important that future studies adapt anxiety interventions for this population [7]. Another population that is not included is children from non-English speaking families. Due to the pilot nature of this study, our first goal was to establish treatment feasibility and efficacy in English-speaking families with the future plan of examining how well this treatment can be translated into other languages. Another point of consideration is that the telehealth model potentially limits the participation of families who may not have the resources to purchase the requisite technology. Although the telehealth model allows families to maintain safety during the COVID-19 pandemic, identification of more inclusive delivery models, such as in-person implementation within local community-based or educational programs, is a priority.

The proposed study is limited by its small sample size and telehealth administration of baseline assessments. However, assessment of treatment feasibility, the primary goal of this study, is unlikely to be impacted by these concerns. Future studies should also include follow-up assessment to evaluate the long-term benefits of the intervention.

Positive findings from this trial would advance the extant literature supporting the feasibility of modified CBT for anxiety in young children with ASD and provide the first empirical support for the integration of treatment strategies directly targeting IU and parental accommodation in anxiety treatments for this population. The next steps would include evaluating DINOSAUR’s efficacy in a large, randomized controlled study featuring an active control group design. Additionally, assessing treatment outcomes utilizing objective markers that are associated with anxiety disorders (e.g., attention bias to threat) would deepen understanding of the biological/cognitive underpinnings of treatment response and potentially lead to the development of neuroscience-based interventions to accompany clinical treatments.

## Conclusion

Anxiety is prevalent in young children with ASD and effective treatments are sorely needed. DINOSAUR is an innovative treatment model for this group that is informed by empirical evidence, targets mechanistic and maintaining factors of anxiety, and utilizes developmentally sensitive intervention methods. Results of this study have important implications for the treatment of anxiety and reduction of long-term psychiatric risk in young children with ASD.

## Data Availability

Not applicable

## Abbreviations

ASD: Autism spectrum disorder
CBT: Cognitive behavioral therapy
IU: Intolerance of uncertainty
